# 828 Awake Caudal Block and Sucrose Pacifier Provides Adequate Analgesia for Excision, Debridement, Grafting of Infant

**DOI:** 10.1093/jbcr/iraf019.359

**Published:** 2025-04-01

**Authors:** Brian Masel, Daniel Arango, Alexis McQuitty

**Affiliations:** Shriners Children’s, Galveston; Shriners Children’s, Galveston; Blocker Burn Unit

## Abstract

**Introduction:**

This is a case series that describes the successful utilization of awake caudal blocks in conjunction with a sucrose pacifier to provide adequate analgesia and anesthesia for extensive excision, debridement, and grafting of a premature infant with primarily 3rd degree, 35% TBSA burns. Neuraxial blocks are very seldom utilized as the primary anesthetic for burn surgery and burn care, and no case reports can be found of utilizing this technique in infants with large TBSA burns. The ability to avoid airway manipulation, apnea inducing narcotics, and dissociative anesthetics in the infant population greatly enhance the safety of anesthetizing and operating on this challenging patient population.

**Methods:**

A 4-week-old, premature 3.5 kg infant presented to our institution after suffering burns from a malfunctioning electrocautery pad while undergoing neurosurgery in another country. The patient suffered 35% TBSA burns encompassing the majority of the area below the T8 dermatome level. Due to the patient’s extensive comorbidities, including hydrocephalus and a high risk of apnea, we chose to avoid a general anesthetic. A pacifier was dipped in a sucrose solution and given to the baby. The baby was then positioned lateral and a caudal block with 1cc/kg of 0.25 % bupivicaine with 1:200,000 epinephrine, 2mg dexamethasone, and 2 mcg dexmedetomidine was administered in the caudal space. The surgery team then proceeded with extensive excision, debridement, and grafting. Throughout the surgery, the infant sucked on the sucrose pacifier. This process was repeated for the next 3 surgeries on this patient.

**Results:**

The patient appeared completely comfortable for the entirety of the 1–2-hour surgeries without any need for supplemental medications either intra or post-operatively. He slept through most of the surgeries or was calmly awake. All vital signs remained stable and he experienced no apnea periods intra or post-operatively.

**Conclusions:**

Awake caudal blocks can safely and reliably provide adequate anesthesia and analgesia for infants with lower extremity and lower trunk burns undergoing acute surgical procedures. This can avoid the need to manipulate the airway and can reduce the risks of post-op apnea.

**Applicability of Research to Practice:**

This technique can be very useful for burn centers, as even large pediatric burn centers are often ill equipped to care for very small infants. This technique provides excellent intra-operative conditions while reducing the risks and level of care needed in the post-op period, when compared with a general anesthetic. Lumbar epidurals, thoracic epidurals, and spinal blocks in acute burn patients of various age groups would be a likely area of future clinical studies.

**Funding for the Study:**

N/A

